# Scottish Vision Group Meeting,Isle of Skye, Scotland,5–7 April 2019

**DOI:** 10.1177/2041669519854233

**Published:** 2019-05-31

## Round Table Discussion: Virtual reality and the future of vision research

**Paul Hibbard^1^, Rafał Mantiuk^2^ and Gizem Rufo^3^**

^1^University of Essex

^2^University of Cambridge

^3^Facebook Reality Labs

## Abstract

Recent advances in consumer-grade virtual reality (VR) equipment have opened up new research topics for vision science. In this discussion session, panellists from a wide range of backgrounds (psychophysics, computer graphics, industry research) will together provide a diverse perspective on VR both as an experimental tool and as a topic of study. Each panellist will give a ten-minute introduction on their work with VR, and these presentations will be followed by a 30-minute round-table discussion session with participation from the audience.

## Keynote Lecture: Integrative active vision

**Iain D. Gilchrist**

University of Bristol

## Abstract

Vision involves *looking* and *seeing:* both are required to support visually guided behaviour. The process of looking involves the generation of saccadic eye movements to point the high-resolution fovea to relevant locations in the environment. The brain network that supports the generation of saccadic eye movements has been studied in detail for over fifty years and we have a detailed understanding of both its anatomy and neurophysiology. What is less clear is how the saccadic network interacts with other brain systems. In this talk I will review examples of our research that attempt to address this issue. I will explore interactions between the saccadic system and face processing, respond timing, salience and choice. Together these studies describe a rich and complex pattern of interactions that allow the brain to generate integrated visually guided behaviour.

## Attentional enhancement of relevant features precedes the suppression of irrelevant features even when distractors are cued

**Plamen A. Antonov, Ramakrishna Chakravarthi and Søren K. Andersen**

University of Aberdeen

## Abstract

Selective attention may allocate limited visual processing resources mainly by enhancing attended or suppressing unattended information. In most experiments, attentional cues indicate to-be-attended rather than to-be-ignored stimuli, thereby potentially biasing selectivity towards enhancement. Here we compared cued shifts of feature-selective attention between conditions in which attended stimuli were cued with conditions in which unattended stimuli were cued. Two superimposed (red or blue), flickering, random dot kinematograms (RDKs) that elicited steady-state visual evoked potentials (SSVEPs) were presented. Auditory cues instructed participants to either attend the relevant (e.g. “attend red”) or ignore (e.g. “ignore blue”) the irrelevant RDK on a trial-by-trial basis and to detect brief coherent motion events in the attended RDK while ignoring such events in the unattended RDK. In ‘attend’ trials, enhancement of SSVEPs elicited by attended stimuli preceded the suppression of unattended stimuli, confirming previous findings. In ‘ignore’ trials, hit rates were lower and stabilized ∼560 ms later post-cue than in ‘attend’ trials. Enhancement still preceded suppression, but importantly, these processes were also shifted back by ∼630 ms. We interpret this as the result of semantically translating “ignore” to “attend” cues instead of using them to directly suppress irrelevant stimuli (e.g. “ignore blue” is substituted by “attend red”). This interpretation is also supported by ERPs elicited by the auditory cues. Overall, our findings suggest that distractor suppression is not under direct voluntary control.

## Gaze contingencies in joint attention as social reward that drives motor learning

**Malgorzata Kasprzyk and Bert Timmermans**

University of Aberdeen

## Abstract

We investigate whether the supposedly rewarding nature of joint attention can drive saccadic motor learning similarly to monetary rewards. Interactionists suggest that social interaction plays a crucial role in development and learning of social skills because seeing others reacting to our actions constitutes an intrinsic reward (Schilbach et al., 2013). Recent studies demonstrated that some forms of social interaction (e.g. joint attention) activate similar brain areas to rewards such as money supporting the notion of this rewarding nature of social interaction (Pfeiffer et al., 2014). However, it is unclear whether social interaction per se can drive learning similarly to those rewards (Schilbach et al., 2010). The current study is an eye-tracking paradigm based on a Milstein and Dorris (2007) study, and requires participants to interact with an anthropomorphic avatar or a cylinder and then conduct a saccade towards a left or right peripheral target. Fast saccades are rewarded by the avatar following participant’s gaze (social reward) or the increase of accumulated gains displayed on the cylinder (monetary reward) with one side being more rewarded then the other (80% vs 20%). We expect that both social and monetary rewards will facilitate faster gaze orientation towards targets with high than low reward probability. If that is the case, it would support the view that the rewarding nature of at least some forms of social interaction facilitate learning (Schilbach et al., 2013) and might explain behavioural differences among autistic individuals whose processing of social interaction is impaired (Pfeiffer et al., 2013).

## Attentional bias reinforces inhibition of return

**Helen Knight^1^, Daniel Smith^2^ and Amanda Ellison^2^**

^1^University of Sunderland

^2^Durham University

## Abstract

Attentional bias is a visual phenomenon wherein certain items are preferentially processed over others in the environment, irrespective of their bottom-up visual properties. It is most commonly present in abnormal populations (GAD, addictions, eating disorders etc.) where psychopathologically-related items capture and hold attention more frequently and persistently than other items in the visual field. Previously, we have found that not only is attentional bias present in said populations, it can also be induced in healthy participants to an arbitrary feature (the colour green, Knight et al., 2016; 2018). This persisted for at least two weeks and affected performance in a change detection task even when the arbitrary visual feature was irrelevant and resulted in poorer performance on the task overall. While this provides substantial evidence that attentional bias influences sensitivity to detect change, its effect on many other attentional paradigms remains unknown. Here, data is presented suggesting that an induced attentional bias impacts on Inhibition of Return – the suppressing of processing stimuli in locations that have previously been attended. Participants engaged in the task used in our previous experiments to induce an attentional bias to the colour green, and then took part in an inhibition of return task where the cue or target could be green. While inhibition of return was observed in all conditions, this was even more extreme in trials where both the cue and target were green, suggesting that attentional bias reinforces inhibition of return. The implication of this for abnormal populations is discussed.

## SSVEP correlates of feature-based attention: a drift-diffusion study

**Nika Adamian and Søren K. Andersen**

University of Aberdeen

## Abstract

Reaction time and responses during visual decision making are well described by models that assume a continuous stochastic accumulation of evidence. Recent studies suggest that steady-state visual evoked potentials (SSVEPs) can explain per-trial evidence accumulation and resulting behavioural decisions. This study aims to test whether the link between SSVEPs and reaction times extends to the cases where both are modulated by feature-based attention. Participants observed two superimposed fields of randomly moving blue and red dots in order to detect coherent motion. When one colour was cued (attended condition), 75% of targets occurred in the attended dot field. When both colours were cued (neutral condition), the targets occurred equally frequently in both dot fields. RT distributions in each condition and stimulus were analysed by estimating three parameters of a shifted Wald model: threshold, shift, and drift rate. Attentional modulation in early visual cortex was quantified by means of SSVEPs in one-second time windows immediately preceding the onset of motion and the subsequent successful detection. Thus, SSVEP epochs and RT data used for the analysis were closely matched. The results of the group-level analysis suggest that attention modulates drift rate and shift parameters of the RT distribution but has little influence on the threshold. The size of the attentional modulation is similar in SSVEPs and drift rates, and is larger than the size of the threshold modulation, suggesting that SSVEPs may reflect the rate of evidence accumulation in a feature-based detection task.

## Spatial attention and awareness in multi-sensory integration

**Matilda Cederblad, Juho Äijälä, Søren Andersen and Arash Sahraie**

University of Aberdeen

## Abstract

Redundancy gain relates to faster responses when multiple sensory stimuli are presented concurrently, in contrast to individual presentations. In last year’s SVG we presented evidence that when awareness was attenuated using continuous flash suppression (CFS) technique, the redundancy gain correlated with the level of subjective awareness. Across multiple experiments we reported that higher awareness led to larger redundancy gains. More recent research on audio visual interactions of stimuli suppressed from visual awareness by CFS has pointed towards a relationship between audio-visual spatial congruency and the degree of visual awareness. In two experiments we have investigated the relationship between both the incidence of aware responses and reaction times to either visual, auditory or combined audio/visual stimuli. In addition we have manipulated the spatial attention by instructing the participants to attend to multiple or a specific spatial location. Our preliminary results suggest that attention plays an important role in awareness of spatially congruent visual/auditory signals.

## Colour appearance in the parafovea as a function of stimulus size

**Ana Rozman and Jasna Martinović**

University of Aberdeen

## Abstract

Colour appearance in the parafovea is a function of stimulus size – very small stimuli appear desaturated compared to larger stimuli (Knau and Werner, 2002, JOSA A). We investigated if this desaturation is equivalent across the lower level mechanisms of colour perception. Participants adjusted perceived contrast for circular colour patches of varying sizes (2˚, 1˚, 0.5˚, 0.33˚, 0.25˚) at varying distances from fixation (4˚ and 5˚) so that it matched perceived contrast of the reference, 2-degree patch. Colours were defined along the cardinal directions of cone opponent colour space (reddish, greenish, bluish, yellowish). A larger departure in the adjustment values was observed for stimuli smaller than 0.33 degrees, as expected based on previous findings. This effect seemed to be equal across the different colour directions. To our knowledge, this is the first replication of the effects reported by Knau and Werner and we will discuss retinal as well as cortical contributions to these observations.

## Luminance and chromatic contrast sensitivity for extended range of light levels

**Maliha Ashraf^1^, Sophie Wuerger^1^, Rafał Mantiuk^2^ and Jasna Martinović^3^**

^1^University of Liverpool

^2^University of Cambridge

^3^University of Aberdeen

## Abstract

Contrast sensitivity functions (CSF) are commonly used to characterise the sensitivity of the human visual system at different spatial scales, but little is known how the CSF changes from the mesopic range to a highly photopic range reflecting outdoor illumination levels.

The purpose of our study was to further characterise the CSF by measuring both achromatic and chromatic sensitivity for background luminance levels from 0.2 cd/m^2^ to 7000 cd/m^2^. Stimuli consisted of Gabor patches of different spatial frequencies and angular sizes, varying from 0.5 to 6 cpd and were displayed on an HDR display with luminance levels up to 15000 cd/m^2^. Contrast sensitivity functions were measured in three directions in colour space, reflecting early post-receptoral processing stages: an achromatic (L+M) direction, a ‘red-green’ (L/(L+M)) direction, and a ‘lime-violet’ direction (S/(L+M)). Within each session, observers were fully adapted to the fixed background luminance (0.2, 2, 20, 200, 2000 or 7000 cd/m^2^).

Our main finding is that the background luminance has a differential effect on achromatic contrast sensitivity compared to chromatic contrast sensitivity. The achromatic contrast sensitivity increases when going to higher background luminance levels up to 200 cd/m^2^ and then shows a sharp decline when the background luminance is increased further. Compared to that the chromatic sensitivity curves do not show a significant sensitivity drop for higher luminance levels. Initial findings imply that our data is not consistent with a local cone contrast adaptation model.

## Exploring the effects of surface properties on hand movements

**Martin Giesel^1^, Anna Nowakowska^1^, Julie M. Harris^2^ and Constanze Hesse^1^**

^1^University of Aberdeen

^2^University of St Andrews

## Abstract

Using a grasping task, we explored how hand movements vary with surface properties. Participants reached over a surface to grasp an object placed on the other side of the surface. We tested five surfaces made from different materials (cardboard, sandpaper, sugar granules, rock salt, AstroTurf). All surfaces had the same size and colour but differed in the granularity and density of their textures. The heights of the surfaces were adjusted to appear similar. The grasping task was performed both under open- and closed-loop viewing conditions. In the open-loop condition, shutter glasses opened at the beginning of a trial but closed as soon as the hand started to move. In the closed-loop condition, the glasses remained open until the end of the movement. The presentation of the different types of surfaces was interleaved, and vision between trials was occluded. We recorded the movements of the index finger, wrist and forearm. After the grasping task, participants rated the roughness, smoothness and pleasantness-to-touch of each surface. To analyse the data, movement parameters were computed relative to those measured for the least rough surface (cardboard) and averaged over observers (N=19). We found that the area under the z-trajectory of the forearm - representing movements of the arm in the vertical direction - closely reflected the roughness ratings for the different surfaces. The area systematically increased with the perceived roughness, suggesting that the rougher the surface, the higher the arm moves over it.

## The contribution of specular highlights to colour constancy

**Rebecca Wedge-Roberts, Marko Nardini, Ulrik Beierholm, Maria Olkkonen and Stacey Aston**

Durham University

## Abstract

Colour constancy – the ability to perceive surfaces as having a constant reflectance under changing illumination – has been proposed to rely on the use of a number of different cues. One cue which has been little studied is specular highlighting on glossy objects, which could potentially be used to estimate the chromaticity of the illuminant. Research into the use of this cue has been inconclusive and suggests that, if present, the effect of this cue is small. We investigated the issue by taking a cue combination approach, which predicts that adding extra cues particularly improves the precision (and not necessarily the accuracy) of perceptual estimates. In a series of experiments, participants were presented with three-dimensional rendered scenes containing either matte or glossy shapes. Participants adjusted the colour of a patch in the scene until it appeared grey. As well as conventional analyses of colour constancy indices and the absolute error in the settings, we examined the variability of settings as a measure of precision. Results so far suggest that adding specular highlights has a small but significant effect on accuracy and colour constancy indices, as well as a less reliable effect on precision. The effect of specular highlights was most prominent when other cues were weakened. Overall, this suggests that people can use specular highlights as a cue to the illuminant but may rely more on other cues when these are present. Further research is required to conclusively determine the situations in which specular highlights are used.

## Colour contrast and colour assimilation in migraine

**Xavier Otazu^1^, Xim Cerda^1^, Olivier Penacchio^2^ and Nilai Sallent^1^**

^1^Universitat Autonoma de Barcelona

^2^University of St Andrews

## Abstract

Several psychophysical studies have found visual dysfunctions in migraine sufferers [1]. Excitatory-inhibitory mechanisms play an important role in colour perception as they control lateral interactions [2]. It has been proposed that facilitation by lateral connections drives colour contrast and suppression by lateral connections drives colour assimilation [3]. Since migraine may be related to a reduced availability of inhibitory activity, we hypothesized that migraineurs would show less assimilation than control subjects. We measured the strength of colour induction in migraine (MO), migraine with aura (MA) and headache-free control (C), with 8 participants in each group. Our results show that there are differences in colour induction between groups, with a stronger colour induction (both contrast and assimilation) in MO than in MA, and in MA than for C. Contrary to our hypothesis, induction was therefore stronger in migraine than in control subjects. We observed that the differences (in the MacLeod-Boynton ‘ls’ colour space) between subject categories were more marked for striped than for uniform stimuli, and in the ‘s’ axis than in the ‘l’ axis, suggesting that the main differences could be found in the koniocellular pathway. These observations also suggest that migraine people have more excitation, which, in turn, leads to an increment of inhibition [4]. Therefore, these results support the idea that there is an excitatory-inhibitory imbalance in migraine.

## 

References[1]O’Hare and Hibbard, Cephalalgia, 2016.10.1177/033310241561895226611680[2]Zaidi et al., Vision Research, 1992.[3]Otazu et al., Journal of Vision, 2010.[4]Nguyen et al., Cephalalgia, 2015.

## Object recognition in deep convolutional neural networks is fundamentally different to that in humans

**Ben Lonnqvist^1^, Alasdair D. F. Clarke^2^ and Ramakrishna Chakravarthi^1^**

^1^University of Aberdeen

^2^University of Essex

## Abstract

Object recognition is a primary function of the human visual system. It has recently been claimed that the highly successful ability to recognise objects in a set of emergent computer vision systems—Deep Convolutional Neural Networks (DCNNs)—can form a useful guide to recognition in humans. To test this assertion, we systematically evaluated visual crowding, a dramatic breakdown of recognition in clutter, in DCNNs and compared their performance to extant research in humans. We examined crowding in two architectures of DCNNs with the same methodology as that used among humans. We manipulated multiple stimulus factors including inter-letter spacing, letter colour, size, and flanker location to assess the extent and shape of crowding in DCNNs to establish a clear picture of crowding in DCNNs. We found that crowding followed a predictable pattern across DCNN architectures that was fundamentally different from that in humans. Some characteristic hallmarks of human crowding, such as invariance to size, the effect of target-flanker similarity, confusions between target and flanker identities, were completely missing, minimised or even reversed in DCNNs. These data show that DCNNs, while proficient in object recognition, likely achieve this competence through a set of mechanisms that are distinct from those in humans. They are not equivalent models of human or primate object recognition and caution must be exercised when inferring mechanisms derived from their operation.

## Building a unitary framework for masking, crowding and grouping: challenges in stimulus design

**Josephine Reuther, Ramakrishna Chakravarthi and Jasna Martinović**

University of Aberdeen

## Abstract

Irrelevant distracters modulate the processing of a target at multiple levels of the visual hierarchy. We aim to investigate whether and how this modulation at early (masking) and mid-level (crowding, contour integration) processing stages relate to each other. However, designing stimuli that can be used to test and link different visual phenomena is notoriously difficult. In our quest to design a common stimulus that can test multiple processing stages, we identified several hurdles in adapting stimuli developed for contour integration tasks to test visual crowding and masking, while maintaining stimulus parameters and the dependent measure across tasks. Traditional contour integration stimuli, such as those in the “snake in the grass” and “snake letter” paradigms, lead to large differences in the range of spatial attention required for a contour integration task, which asks participants to integrate over a big area, and tasks that ask participants to base their judgement on a single item, as in masking and crowding paradigms. Furthermore, in their original form, these contour integration stimuli would either impose high levels of spatial uncertainty or require “snake-detection” before item individuation when used to measure crowding or masking. Here we will present a stimulus that not only minimizes these issues, but also incentivises participant behaviour (integrate vs individuate) in line with the phenomenon of interest.

## Clarity of text and comfort of reading

**Arnold Wilkins**

University of Essex

## Abstract

Two simple algorithms that assess the spatial periodicity of text can predict respectively the comfort and the speed with which text is read. Fonts differ considerably. Ratings of discomfort from a wide variety of images are predictable from the extent to which the images are “un-natural”, as assessed by fitting a 1/f cone to the two-dimensional Fourier transform. The size of the residuals in such a fit predicts discomfort from text, as reflected in the choices people make when adjusting i-Books. Reading speed can be predicted from the first peak in the horizontal autocorrelation of text. The higher the peak, the lower the reading speed because more time is required to re-align the eyes following a saccade. The effects on comfort and reading speed may be related to cortical hyperexcitability and a consequent susceptibility to the spatial periodicity of text. The two algorithms provide measures that are largely independent and can guide the visual design of written material in various orthographies, including Hindi and Chinese ideograms.

## Effects of facial expression on expression detection and contrast sensitivity: an extension of Hedger, Adams and Garner (2015)

**Abigail Webb and Paul Hibbard**

University of Essex

## Abstract

Fearful faces are associated with a number of perceptual advantages. Recent accounts posit that these fear biases are driven by the low-level image properties belonging to fearful faces. In particular, Hedger and colleagues (2015) show that fearful expressions are especially high in effective contrast; the extent to which their Fourier amplitude is matched to the contrast sensitivity function. This effective contrast was also a significant predictor of fearful faces’ detectability during a backward-masking paradigm. Importantly, images used for image analyses and a backward-masking paradigm were facial stimuli that had been normalised for RMS contrast. Stimuli normalised for physical contrast are not necessarily equal at the subjective level (perceived contrast) (O’Hare & Hibbard, 2011), and Menzel and colleagues (2018) demonstrate natural expression-related differences between expressions’ low-level image properties that are hindered during stimulus normalisation. The present study performs a behavioural investigation of Hedger’s (2015) image analyses with a contrast sensitivity task that uses facial stimuli. Visual thresholds do not differ according to raw (non-normalised) facial expressions. A replication of Hedger’s (2015) analysis of images’ effective contrast also shows that higher effective contrast in fearful expressions is significantly influenced by expressions having first been contrast normalised, compared to when they undergo analysis in their raw, normal format. Finally, a backward-masking study using both raw and RMS normalised faces shows that while contrast normalisation does indeed influence expression detection, it does not determine the bias for fear expressions.

## The sun illusion in a medieval Irish Astronomical Tract

**Helen Ross**

University of Stirling

## Abstract

Early medieval Irish scholars were famous for their knowledge of classical texts and astronomy, later authors less so. The Irish Astronomical Tract is a 14th-15th century Gaelic document, based mainly on a Latin translation of the Jewish astrologer Messahala (8th-9th century). The Irish text has been translated into English by John Williams (The Irish Astronomical Tract: A case study of scientific terminology in 14th century Irish. M.Phil Thesis, University of Sydney, 2002). It contains a passage about the sun illusion - the apparent enlargement of celestial bodies when near the horizon compared to higher in the sky. This passage occurs in chapter 7, entitled “The rotundity of the earth and the knowledge of day and night”. Here the author denies that the change in size is caused by a change in the sun’s distance, and instead ascribes it (incorrectly) to magnification by atmospheric vapours, likening it to the bending of light when looking from air to water or through glass spectacles. This section does not occur in the Latin version of Messahala. The Irish author may have based the vapour account on Aristotle, Ptolemy or Cleomedes, or on later authors that relied on them. He seems to have been unaware of alternative perceptual explanations offered by these and other authors. The Tract does not tell us much about the state of late medieval Irish science, except that Irish scholars remained in touch with some aspects of mainstream science. The refraction explanation persists today in folk science.

## Ocular equivocation: the rivalry between Wheatstone and Brewster

**Nick Wade**

University of Dundee

## Abstract

Ocular equivocation was the term given by Brewster in 1844 to binocular contour rivalry seen with Wheatstone’s stereoscope. The rivalries between Wheatstone and Brewster were personal as well as perceptual. In the 1830s both Wheatstone and Brewster came to stereoscopic vision armed with their individual histories of research on vision. Brewster was an authority on physical optics and had devised the kaleidoscope; Wheatstone extended his research on audition to render acoustic patterns visible with his kaleidophone or phonic kaleidoscope. Both had written on subjective visual phenomena, a topic upon which they first clashed at the inaugural meeting of the British Association for the Advancement of Science in 1832 (the year Wheatstone made the first stereoscopes). Wheatstone published his account of the mirror stereoscope in 1838; Brewster’s initial reception of it was glowing but he later disputed Wheatstone’s priority. They both described investigations of binocular contour rivalry but their interpretations diverged. As was the case for stereoscopic vision, Wheatstone argued for central processing whereas Brewster’s analysis was peripheral and based on visible direction. Brewster’s lenticular stereoscope and binocular camera were described in 1849. They later clashed over Brewster’s claim that the Chimenti drawings were made for a 16th century stereoscope. The rivalry between Wheatstone and Brewster is illustrated with anaglyphs that can be viewed with red/cyan glasses; the anaglyphs include rivalling ‘perceptual portraits’ as well as examples of the stereoscopes and stimuli used to study ocular equivocation.

## Cue recruitment for judgements of motion-in-depth

**Lauren Murray and Ross Goutcher**

University of Stirling

## Abstract

In virtual environments, perceptual errors may be overcome through the provision of additional stimulus information. In two experiments, we explored whether the visual system can use such information through recruitment of a novel metric colour cue to motion-in-depth. Participants were presented with fronto-parallel checkerboard surfaces, with motion-in-depth defined by changes in both binocular disparity and image size. Colour information was added by correlating stimulus colour with position in depth along a red-blue gradient. In Experiment 1, we varied the relative amplitude of changing disparity and changing size cues to measure the conflict required to elicit a perception of changing object size. In Experiment 2, we used a perturbation analysis method to measure the perceived speed-in-depth of a stimulus where changing disparity and changing size cues were in conflict by a fixed amount, differing in speed by 4.8cm/s. In each experiment, the novel colour cue correlated with either the disparity or size cue on a trial-by-trial basis. It was expected that the colour correlated cue should receive more weight and therefore bias participants’ judgements. In Experiment 1, thresholds for the perception of changing object size indicated an increase in task difficulty when the colour cue was correlated with changing image size. No effect of colour was found in Experiment 2. Our results suggest that cue recruitment is task dependent but may aid motion-in-depth judgements where stimulus information is ambiguous. We also consider possible roles for attentional factors in the recruitment of novel cues.

## Head movements induced by visual stimulation in virtual reality

**Katharina MT Pohlmann, Louise O’Hare, Adrian Park and Patrick Dickinson**

University of Lincoln

## Abstract

Visual discomfort and cybersickness are often experienced when viewing virtual environments through a head-mounted display (HMD). Motion-in-depth in particular is believed to result in increased visual discomfort compared to lateral motion. The visual and vestibular-system are closely connected. An illusory motion from a visual stimulus can lead to uncertainty in the vestibular system. This conflict can lead to motion sickness and postural instability and is measurable in the extent of head movements. This study examines the relationship between head movements and discomfort induced by illusory motion from a visual stimulation. Observers viewed expanding (motion-in-depth) and rotating (lateral) motion illusions through an Oculus Rift HMD. They rated illusion strength and their experienced discomfort levels (headache, dizziness, eye strain, and blurred vision) for each stimulus. Preliminary results indicate a positive relationship between illusion strength and experienced discomfort. Rotating illusions were rated as stronger than expanding illusions but no significant difference in discomfort ratings between them was found. This indicates that motion illusions that elicit motion-in-depth cause more discomfort in observers than motion illusions eliciting lateral motion. Sway magnitude was stronger in the antero-posterior direction than in the medio-lateral direction. We also expect expanding motion illusions to lead to more antero- posterior head movements compared to stimuli exhibiting a rotating motion illusion. We conclude that motion illusions eliciting motion-in-depth cause more discomfort in observers than motion illusions eliciting lateral motion which is represented in more postural swaying particularly in the antero-posterior direction.

## Waveform structure limits sensitivity to cyclopean form

**Ross Goutcher**

University of Stirling

## Abstract

The perception of binocular disparity-defined three-dimensional form depends upon the successful measurement of these disparities, together with the measurement of how they vary across local image regions. Sensitivity to such disparity-defined, cyclopean form may thus be set at both the level of early, absolute disparity measurements, defined in retinal co-ordinates and later, in the encoding of relative disparities (i.e. differences in disparity across space). To examine the contribution of each of these factors, in a series of experiments, we measured additive noise thresholds for cyclopean orientation discrimination in a range of disparity-defined random-dot waveforms, including square, sine, triangular and sawtooth waves. Participants were presented with random-dot waveforms at ±20 degrees from vertical and asked to judge whether the stimulus was clockwise or counter-clockwise rotated. Noise was added to the stimuli in the form of random Gaussian disparity noise, allowing for the measurement of thresholds for the standard deviation of noise required to impair orientation discrimination performance. Thresholds were higher for squarewave stimuli than for other waveforms, indicating an increased tolerance for disparity noise. We consider these results in terms of the cross-correlation-based measurement of absolute disparity and the responses of disparity-frequency tuned hypercyclopean channels.

## Adding motivation and feedback does not facilitate optimal eye movement strategies

**Warren James^1^, Josephine Reuther^1^, Ellen Angus^1^, Alasdair Clarke^2^ and Amelia Hunt^1^**

^1^University of Aberdeen

^2^University of Essex

## Abstract

Participants are given a choice about where to fixate to detect a target that is equally likely to appear in one of two possible target locations. When the locations are close together, the best choice is to fixate between them. When the two locations are too far apart to be visible from a central location, a better strategy is to fixate one target location or the other. Despite the existence of this simple strategy to maximize detection accuracy, participants consistently fail to modify their choice with the distance between the targets. One possible reason for this failure is a lack of incentive to be accurate or clear feedback on detection performance. Here we provided salient feedback to a virtual character (a penguin), contingent on participant’s performance. This functioned as explicit feedback for the participant to be able to monitor their own performance on a trial by trial basis, as well as an effective motivator of detection accuracy (according to participants’ reports). Despite this, fixation strategies were not improved. The results argue against the hypothesis that participants’ poor decisions result from a lack of motivation to perform well. In line with previous research, participants tend to default to a variable pattern of behaviour, rather than discerning an over-arching strategy to maximise success.

## Search strategies vary between different types of stimuli

**Anna Nowakowska^1^, Alasdair Clarke^2^, Josephine Reuther^1^ and Amelia Hunt^1^**

^1^University of Aberdeen

^2^University of Essex

## Abstract

Nowakowska, Clarke and Hunt (Proc. Royal. Soc. B, 2017) tested the prediction that eye movements are directed to locations that yield the most information. We split search arrays into two halves vertically, with homogeneously-oriented line segments on one half and heterogeneously-oriented line segments on the other. When the target (a line segment oriented 45˚ to the right) was present on the homogenous side, it could be easily detected using peripheral vision, so observers should only make fixations on the heterogeneous side. However, we found that most participants over-fixated the homogeneous half, at a substantial cost to reaction time. When we replaced line segments with desktop application icons, with a variable set on one (heterogeneous) side and uniform folders on the other (homogenous) side, we observed uniformly efficient search behaviour across all participants. This efficient behaviour was preserved when we pixelated the icons to make them unrecognizable, and for greyscale version of the icons, suggesting familiarity and colour are not driving efficient search. When the icons were replaced with polygons rotated on the heterogeneous side and aligned in straight rows on the homogenous side, search became less efficient. Having ruled out several possible explanations for the striking differences in search strategies between different classes of stimuli, we will discuss what explanations remain.

## Performance on a global motion task cannot effectively distinguish between poor and good readers: results from a cross-sectional study of primary-aged children

**Barbara Piotrowska and Alexandra Wills**

Edinburgh Napier University

## Abstract

Although primarily conceptualised as a disorder of phonological processing, developmental dyslexia (DD) is often associated with broader problems perceiving and attending to transient or rapidly-moving visual stimuli. However, the extent to which such visual deficits represent the cause or the consequence of dyslexia remains contentious, and very little research has examined the relative contributions of phonological, visual, and other variables to reading performance more broadly. We measured visual sensitivity to global motion (GM) and global form (GF), performance on various language and other cognitive tasks believed to be compromised in DD together with a range of social and demographic variables often omitted in previous research, such as age, gender, non-verbal IQ (NVIQ), and socio-economic status (SES) in an unselected sample (n = 132) of children aged 6-11.5 yrs from two different primary schools in Edinburgh, UK. We found that: (i) GM sensitivity (but not GF) was significantly lower in poor readers, but effect size was medium; (ii) GM sensitivity accounted for only 3% of the variance in reading scores; (iii) GM sensitivity deficits were only observed in 16% of poor readers; (iv) the best predictors of reading performance were phonological awareness, NVIQ, and SES, suggesting the importance of controlling for these in future studies of vision and reading. Case-wise analysis of the visual and cognitive deficits in poor readers revealed a very mixed picture, with no clear clusters of difficulties. These findings suggest that developmental dyslexia is unlikely to represent a single category of neurodevelopmental disorder underpinned by lower-level deficits in visual motion processing.

## The effect of feedback and stimulus presentation protocol on perceptual learning for local and global motion and form

**Jordi Asher and Paul Hibbard**

University of Essex

## Abstract

The necessity for external reinforcement, during perceptual training is a topic of much debate. Learning has been found to occur with and without feedback or by interleaving easy and difficult trials. To investigate this in detail we performed a large multi-level experiment, using interleaved easy and difficult trials, varying the type of training task (Motion or Form), the level of processing (Local or Global), presence of feedback (With or Without) and finally the method of stimulus presentation (Adaptive staircase (QUEST) or Method of constants (MOCS)). Learning was robust and occurred for both feedback groups whether using an adaptive staircase or method of constant stimuli. Some differences were identified between the MOCS and QUEST trained results in that at lower stimulus intensities QUEST methods elicited better performance (when there was No Feedback). At higher stimulus intensities MOCS trained groups performed the same or better than QUEST trained groups. This suggests that when there is a high level of external noise in the stimulus, the internal signal provided by the presence of easy exemplars, at set thresholds facilitated learning when trained on QUEST, but not when trained with randomly interleaved intensities using MOCS. Interleaving high and low accuracy trials for both local and global tasks leads to perceptual learning independent of external feedback.

## Neural correlates of motion perception

**Danai Papadaki^1^, Karin S. Pilz^2^ and Rama Chakravarthi^1^**

^1^University of Aberdeen

^2^University of Gronigen

## Abstract

Motion perception is an essential visual skill which aids our navigation through the environment, allows us to identify self and object motion, and enhances our social interactions. Previous research has demonstrated an advantage for visual processing along the cardinal directions (i.e., horizontal and vertical) compared to oblique (i.e., diagonal) directions. This so called “oblique effect” has been documented in detail for orientation discrimination. The oblique effect for motion direction discrimination has received far less attention. In the present experiments, we systematically assessed the oblique effect in motion perception for motion detection and discrimination and investigated the underlying mechanisms of directional anisotropies using electroencephalography. In one experiment, participants were asked to detect coherent motion from random dot kinematograms (RDKs), and in a second experiment were asked to indicate the direction of motion from RDKs. Reaction times and accuracy were measured as well as latencies and amplitudes of event related potentials. We tested differences among these measures between (and within) cardinal and oblique motion directions. Whereas neural differences between motion directions are subtle, behavioural results show that the oblique effect is more pronounced for motion discrimination than detection. This is in line with previous studies showing that the oblique effect is highly dependent on the tasks. Results will be discussed within the context of the prevalence and relevance of motion directions in our visual environment.

## Active 2D & 3D tasks for exploring the nature of MIPS

**Ian M. Thornton and Joseph Muscat**

University of Malta

## Abstract

Motion-induced position shifts (MIPS) refer to situations in which the global, physical position of a target object is misperceived due to its own local motion. In a recent series of studies, we have been exploring whether giving observers active control of the global position of a target object modulates the size of these illusions, relative to more traditional passive judgements. Here, we present new data from a 2D task, where a target Gabor patch had to be steered through a slalom course of gates. We measured deviations from the gate centres as a function of local drift direction. Gate entry was strongly influenced by the direction of local drift. These active errors did not change as a function of time-on-task and were considerably larger than those predicted from a comparable passive task, an effect that did not relate to poor motor control. We also present data from a new study exploring MIPS in a scenario that simulates 3D motion in depth.

## Attentional facilitation of tracked targets limits multiple object tracking performance

**Søren K. Andersen, Rafael Lemarchand and Nika Adamian**

University of Aberdeen

## Abstract

The ability to keep track of moving objects is commonly investigated using the multiple object tracking paradigm (MOT). Whereas it was initially proposed that MOT performance is afforded by about four pre-attentive tracking mechanisms operating in parallel, more recent work has demonstrated that tracked targets are attentionally enhanced in early visual cortex. Thus selective attention is the most likely limiting factor for MOT performance. However, the magnitude of attentional enhancement in a previous study was independent of the number of tracked objects, which complicates this interpretation. Here we measured the magnitude of attentional enhancement of tracked targets in an MOT task using steady-state visual evoked potentials (SSVEPs). In different conditions, participants tracked two, four or six out of twelve objects. Unlike the previous study, trials were physically identical between conditions, except for the initial cue. Under these conditions of tight control of possible physical stimulus confounds, we found a consistent pattern of decreasing attentional enhancement of SSVEP amplitudes with increasing set-size of tracked objects.This finding supports the idea that limitations of attentional selection underlie the decrease of MOT performance with increasing set-size. It is also consistent with our recent proposal, that concurrently selecting multiple feature values (here: object locations) within the same feature dimension reduces the magnitude of attentional selection, whereas selecting multiple features of different dimensions does not.

## Target-tracking: exploring the effects of prolonged exposure to feedback delay in virtual reality

**Ida Nilsen and Loes van Dam**

University of Essex

## Abstract

In Virtual Reality (VR) feedback delays between actual hand movements and when the movements are represented visually, lead to reduced subjective ratings of ownership, agency and presence. However, using a target-tracking task we have previously shown that with prolonged exposure to the delay the subjective experience in VR again improved (van Dam & Stephens, PLOS ONE, 2018). Here, we investigated whether this improvement in subjective experience is due to genuine delay adaptation, i.e. a shift in the timing of the movements, or the result of a general improvement in the target-tracking task over time. To this end we used two different target-tracking conditions: 1) target movement was unpredictable, in which case delay adaptation is impossible; 2) target movement was predictable by presenting the movement trajectory ahead of time, in which case delay adaptation should occur (Rohde, van Dam & Ernst, JOV, 2014). In both conditions a 200 ms feedback delay was added during the exposure phase of the experiment. Behavioural tracking results and subjective ratings of ownership, agency and presence were recorded for pre and post-test trials without a delay and during the exposure phase with the delay. Our results showed that genuine delay adaptation occurred only for the predictable target (confirming Rohde et al., 2014) whereas the spatial error decreased in both cases. However, the subjective ratings for ownership, agency and presence improved for both predictable and unpredictable targets. This suggests that the improvement in subjective ratings with prolonged exposure to the delay does not require genuine delay adaptation.

## Learning and combining novel perceptual cues

**Stacey Aston, Marko Nardini and Ulrik Beierholm**

Durham University

## Abstract

New technologies offer opportunities to enhance human perceptual abilities. Imagine a surgeon who can hear their way around the human body when performing keyhole surgery using a novel auditory signal, or a driver who can feel distances to nearby objects using a novel tactile signal. Making use of these technologies requires an understanding of the limits of human abilities to acquire and use novel cues. Can novel cue use share all the characteristics of native cue use? One such characteristic is cue combination, where multiple cues to the same world property are combined, increasing precision in perceptual estimates. In a series of experiments we find that while participants are able to learn to use a variety of novel visual cues to location (colour, angle size, shape, line length for 1D estimation), whether or not they begin to combine the novel cues with a native (spatial) cue depends on duration of exposure and is an unreliable effect easily abolished by adding a second novel cue to the experiment or by learning two novel cues in succession (the first is combined, the second is not). In conclusion, while perceptual and cognitive systems can quickly learn to use novel cues to location, learning to combine the novel cues with native cues is a longer process and is sub-optimal compared to a Bayes-optimal prediction (within the time frame of these experiments). To better understand the limits of cue combination with new cues, we need to develop models that account for these seeming sub-optimalities.

## Age-related differences in the use of distal landmarks during object-location encoding

**Vladislava Segen^1^, Marios Avraamides^2^, Timothy Slattery^1^ and Jan Wiener^1^**

^1^Bournemouth University

^2^University of Cyprus

## Abstract

The ability to form spatial representations of the environment is a prerequisite for successful navigation. Our previous research suggests that there are age-related differences in gaze behaviour during spatial encoding. Specifically, older adults’ gaze behaviour during spatial encoding was more dispersed, suggesting that they are attending distal information more often than young adults who exhibit a preference for local cues. However, it is unclear whether older adults are using the distal room cues to facilitate performance or if they are distracted by them. This study examined whether the presence of distal cues improves older adults ability to encode the spatial locations of objects. During the learning phase participants viewed arrays of 6 identical objects placed in the middle of a rectangular virtual room. The room either contained informative, uninformative (identical) or no distal cues. During the test phase participants indicated whether the spatial positions of the objects in the room have changed. The room was viewed either from the same viewpoint or from a different perspective than in the learning phase. Gaze behaviour was recorded to assess the visual strategies used by older and younger adults. Region of interest analysis as well as stimuli-independent gaze analysis will be used to explore the spatial coding strategies used by younger and older adults.

## Neural correlates of spatial bias in healthy cognitive ageing

**Monika Harvey, Gesine Maerker, Gemma Learmonth and Gregor Thut**

University of Glasgow

## Abstract

Young adults tend to overestimate the size and luminance of objects located in the left side of space(“pseudoneglect”), a spatial bias deemed to be caused by a right hemisphere dominance for visuospatial attention. Intriguingly, for some spatial tasks, healthy older adults have been shown to lose this leftward bias, yet at present little is known as to whether these behavioural shifts reflect hemispheric changes. Here we present two experiments: firstly we aimed to identify a spatial task teasing out age related spatial bias changes. Secondly, we wanted to investigate potential hemispheric alterations with EEG. In the first experiment we found that for a single given task, both young and older participants showed consistent spatial biases across different testing days. However, different tasks generated different biases, with only the landmark task (in which participants are instructed to indicate which side of a pre-transected centrally presented line is shorter/longer) showing significant age related bias shifts. In the second experiment, we compared young and older adults on this task whilst recording event-related potentials (ERPs). Full-scalp cluster mass permutation tests identified a larger right parieto-occipital response for long compared to short landmark stimuli in young adults, an effect not present in the older group. To conclude we report task and stimulus-driven reduction of right hemispheric control over spatial attention in older adults. Future studies will need to determine whether these hemispheric changes can be mapped for other spatial tasks and methodologies, and whether they represent normal aging processes or an early indication of neurodegeneration.

## The timing of motion

**Marlene Poncet and Justin Ales**

University of St Andrews

## Abstract

When two objects are presented at two locations successively, they are seen as a single object moving from one location to the other. Behavioural studies have shown that the smaller the time delay between two presentations, the stronger the motion percept. Interestingly, the reason for this finding remains unknown. Here, we assessed this by recording participants’ Steady-State Visual Evoked Potentials (SSVEP) for stimuli flashed periodically at different frequencies (2.6 Hz, 5.2 Hz, 10.4 Hz). To test for temporal effects, we manipulating the duty-cycle, that is, the proportion of time that the stimulus was presented during a cycle. The stimulus was presented either at the same location (flicker condition) or at two alternating locations (moving condition). Our results show that at 2.6 and 5.2 Hz, increasing duty-cycle decreases SSVEP amplitudes. We also find that the perception of motion increases as a function of duty-cycle for moving and, unexpectedly, for flickering stimuli. Importantly, SSVEP amplitudes are inversely correlated with motion perception irrespective of whether the stimulus is actually changing position. On the other hand, at 10.4 Hz, both SSVEP amplitudes and motion perception are not affected by duty-cycle. Further analyses reveal that stimulus energy cannot account for our findings. In conclusion, our study shows that for both static and moving stimuli, increasing duty-cycle increases the strength of motion perception and decreases brain responses. This suggests that temporal interactions at the neural level play a crucial role in creating a moving percept.

## Cross-modal effects of preceding distractors on perceived duration of visual and auditory stimuli

**Martin Lages^1^ and Franziska Klein^2^**

^1^University of Glasgow

^2^University of Oldenburg

## Abstract

We investigated whether the perceived duration of a visual or auditory test stimulus depends on preceding distractors from the same or a different modality. In each trial a participant first perceived a reference stimulus of 500 ms. After an interval five short distractors or a single long distractor was presented immediately followed by a test stimulus. In randomly intermixed trials we varied the distractors (long, short) and the duration of the test stimuli (300 to 700 ms in steps of 50 ms). In four separate blocks of trials, administered in two sessions over consecutive days, we tested twelve participant in visual-only, auditory-only, visual-auditory and auditory-visual conditions. Ten participants managed to reliably discriminate between the duration of the reference and test stimuli in this 2IFC task. The results of logistic linear mixed-effect models suggest that participants systematically overestimated the duration of the test stimuli in the context of the short distractors and underestimated duration of the test stimuli in the context of the long distractors. The effect of the distractors was relatively strong in both uni-modal conditions but weak in the two cross-modal conditions. Individual variability in discrimination performance and interactions between distractor and stimuli are discussed.
